# Intratracheal interleukin‐10 administration reduces acute lung injury induced by sulfur dioxide exposure in mice

**DOI:** 10.14814/phy2.71079

**Published:** 2026-08-25

**Authors:** Amy J. Nelson, Todd A. Wyatt, Bradley W. Richmond, Belinda Diaz, Angela M. Gleason, Michael J. Duryee, Geoffrey M. Thiele, Ted R. Mikuls, Jill A. Poole

**Affiliations:** ^1^ Division of Allergy & Immunology University of Nebraska Medical Center Omaha Nebraska USA; ^2^ Division of Pulmonary, Critical Care & Sleep University of Nebraska Medical Center Omaha Nebraska USA; ^3^ Veterans Affairs Nebraska‐Western Iowa Health Care System, Research Service Omaha Nebraska USA; ^4^ Department of Environmental, Agricultural and Occupational Health, College of Public Health University of Nebraska Medical Center Omaha Nebraska USA; ^5^ Division of Allergy, Pulmonary, and Critical Care, Department of Medicine Vanderbilt University School of Medicine Nashville Tennessee USA; ^6^ Department of Veterans Affairs Vanderbilt University School of Medicine Nashville Tennessee USA; ^7^ Division of Rheumatology & Immunology, Department of Internal Medicine, College of Medicine University of Nebraska Medical Center Omaha Nebraska USA

**Keywords:** chemical, inflammation, injury, lung, treatment

## Abstract

Therapies capable of reducing acute high‐consequence airborne chemical exposure‐induced lung disease are lacking. This study seeks to determine the therapeutic potential of interleukin (IL)‐10 administered following acute sulfur dioxide (SO_2_) exposure in mice. C57BL/6 male and female mice were exposed to SO_2_ gas at 125 ppm for 4 h and subsequently treated 2–3 h later with IL‐10 (1 μg) or vehicle control by intratracheal instillation for a total of 3 doses over 2 days with comparisons to sham‐treated mice (*N* = 20 mice/group). IL‐10 treatment reduced SO_2_‐induced weight loss with corresponding reductions in SO_2_‐induced circulating markers of inflammation (pentraxin‐2 and fibrinogen). IL‐10 reduced SO_2_‐induced markers of lung cell permeability (surfactant protein‐D and protein), and cell death (lactate dehydrogenase [LDH]). SO_2_‐induced lung infiltrates of neutrophils and activated macrophages were reduced with IL‐10 treatment. IL‐10 reduced SO_2_‐induced increases in total protein, Substance P, apoptotic cells, post‐translational citrullinated and malondialdehyde‐acetaldehyde‐modified proteins, and vimentin. Compared to female mice, SO_2_ exposure led to greater increases in serum pentraxin‐2, fibrinogen, LDH levels, and lavage fluid cells in males. In conclusion, short‐term, intratracheal IL‐10 therapy administered after acute SO_2_ exposure favorably modulates acute lung injury consequences to suggest a therapeutic potential to decrease adverse systemic and lung effects.

## Graphical abstract

Intratracheal IL‐10 administration following acute sulfur dioxide exposure reduces sulfur dioxide‐ induced systemic and lung injury and inflammatory consequences in mice.

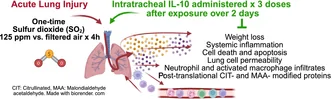

## 1 INTRODUCTION

Inhalant hazardous chemicals represent high‐consequence public health threats, as they induce acute lung injury and inflammation leading to the development of chronic lung diseases, including asthma, chronic obstructive pulmonary disease, pneumonitis, and fibrosis (Marzec & Nadadur, 2024). Although efforts to reduce exposure and promote the appropriate use of respiratory protective equipment are vitally important, there are currently no medical therapies available to ameliorate chemical‐induced lung injury and inflammation without exerting untoward side effects (Marzec & Nadadur, 2024).

Recognizing that there are over 200 chemicals/gases of concern, sulfur dioxide (SO_2_) is an important gaseous air pollutant, one of six pollutants comprising the air quality index, with links to adverse human health effects (Khalaf et al., 2024). High‐concentration SO_2_ has been implicated in inducing acute lung injury and inflammation with potential progression to chronic lung disease, for which preclinical animal modeling has been established (Gutor et al., 2024). SO_2_ is an industrial air pollutant sourced from power plants, smelters, and coal burning processes, as well as via the production of fertilizers, paper, and food preservatives. SO_2_ is also found in burn pits, military operations, agriculture emissions, and is often detected in disaster events (Marzec & Nadadur, 2024). Although mechanistic understanding of acute SO_2_‐induced lung injury and inflammation remains limited, SO_2_ dissolves in airway surface liquid to form sulfurous acid, which induces tissue injury leading to epithelial barrier disruption, rapid bronchoconstriction, recruitment of inflammatory cells, and airway edema (Stauffer & Tat, 2026). Repetitive exposure to SO_2_, as a model of burn pit exposures in mice, led to airway wall remodeling, adaptive immune cell activation, and pulmonary vascular disease with an increase in oxidative stress pathways (Gutor et al., 2024). Extremely high SO_2_ exposures (>100 ppm) (National Institute for Occupational Safety and Health, 2014) for short durations have been recognized as dangerous and can result from accidents/spills originating from domestic or international sulfur plants, oil fires during the Gulf and Iraq Wars, and volcanic eruptions (www.va.gov/health‐care/health‐needs‐conditions/chemical‐hazardous‐materials‐exposure/, www.osha.gov, www.epa.gov). In general, high‐consequence chemical exposures, such as those resulting from natural disasters, catastrophic incidents, or other accidents, can set the stage for progressive lung disease (Krefft et al., 2015; Morimoto et al., 2021; Reid & Maestas, 2019; Robinson et al., 2011). Symptomatic management by means of oxygen, bronchodilators, and sometimes corticosteroids can improve patient quality of life following such exposures; however, there are no interventions capable of hastening lung pathologic recovery and halting progression to chronic lung disease (Tarlo, 2012).

Interleukin (IL)‐10 represents a potential efficacious intervention post‐SO_2_ exposure, due to its ability to mitigate tissue damage, improve wound repair and fibrosis, and promote recovery (Steen et al., 2020). We have demonstrated several striking beneficial effects with administration of unmodified, recombinant IL‐10 as a direct lung‐delivered (as opposed to systemic‐delivered) therapy after exposure to high‐concentration endotoxin and complex organic dust extracts in preclinical animal modeling studies, avoiding potential systemic side effects (Poole, Gaurav, et al., 2022; Schwab et al., 2025; Schwab, Wyatt, Nelson, et al., 2024). Short‐term (3 doses over 2–3 days) lung‐delivered IL‐10 significantly reduced influx of inflammatory cells (i.e., neutrophils, monocytes, and macrophages), pro‐inflammatory and pro‐fibrotic mediators, adverse lung pathology, and airway dysfunction induced by endotoxin and/or organic dust extract (Poole, Gaurav, et al., 2022; Schwab et al., 2025; Schwab, Wyatt, Nelson, et al., 2024). Moreover, IL‐10 therapy reduced the expression of post‐translational protein modifications formed during oxidative stress and (i.e., malondialdehyde‐acetaldehyde (MAA) and citrulline (CIT)) (Schwab et al., 2025) that contribute to chronic disease development (Duryee et al., 2021; England et al., 2019; Samara et al., 2017).

The value of IL‐10 as a therapeutic countermeasure against acute chemical exposures such as SO_2_ remains unknown and is the objective of this study. These proof‐of‐concept studies could have an important impact in laying the pre‐clinical groundwork for understanding how short‐term IL‐10 regulates acute chemical‐induced acute lung injury to ultimately develop new strategies to treat harmful public health or military events or accidents involving the release of toxic chemicals, without inducing untoward systemic effects.

## 2 MATERIALS AND METHODS

### 2.1 Mouse model

C57BL/6 male and female mice (age 6–8 weeks) were purchased from The Jackson Laboratory (Bar Harbor, ME). All mice were housed in specific pathogen‐free facilities maintained at 20°C–24°C with a 40% relative humidity and a 12‐h light/dark cycle. All mice had ad libitum access to standard rodent chow (Teklad Rodent Diet 7012/LM‐485, Inotiv, Indianapolis, IN) and filtered tap water. The study was conducted and reported in accordance with ARRIVE guidelines (https://arriveguidelines.org). All animal protocols were reviewed and approved by the University of Nebraska Medical Center (UNMC) (Omaha, NE) Institutional Animal Care and Use Committee (IACUC) and were in accordance with NIH guidelines for the use of rodents. This research was conducted under a valid license or permit from the UNMC IACUC.

**FIGURE 1:**
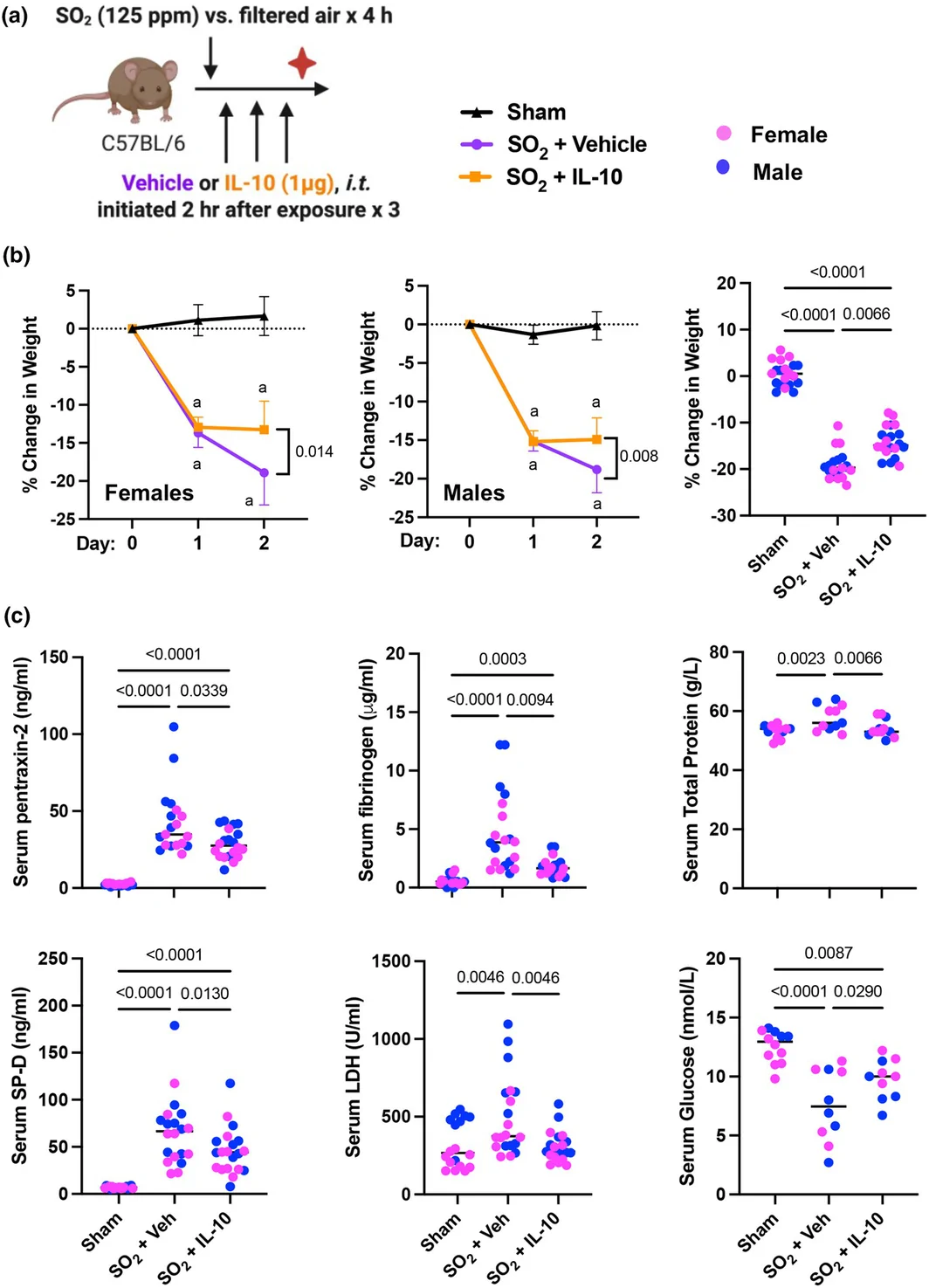
Lung‐delivered IL‐10 administered after acute SO_2_ exposure reduces SO_2_‐induced weight loss, systemic inflammatory responses, lung permeability, and cell death, and improves glucose consumption. (a) Mice were treated with SO_2_ (125 ppm) or filtered air × 4 h and received either treatment with IL‐10 (1 μg) or vehicle (Veh) initiated 2–3 h later for a total of three doses over 2 days and euthanized at 5 h after final treatment. (b) Line graph depicts sex‐based weights over time with scatter dot plot demonstrating percent change in weight upon study completion across indicated groups. (c) Serum levels of acute phase protein pentraxin‐2 and fibrinogen (systemic inflammation); total protein and serum surfactant protein‐D (SP‐D) (lung permeability); lactate dehydrogenase (cell injury/death) (LDH); and glucose. All data expressed as mean of *n* = 20 mice/group (10 female, magenta; 10 male, blue), with values less than 20 due to limited sampling. Statistically significant *p* values denoted by line.

SO_2_ exposure (vs. filtered air) was performed in the animal care facility using an inExpose inhalational exposure system (SCIREQ, Montreal, Canada) approved by the IACUC and the Institutional Biosafety Committee (IBC). The system was operated under a standard fume hood, and ppm was controlled with Portable SO_2_ Gas Detector, 0 to 1000 ppm (Sisco, Guilin, China). Modifying prior SO_2_ animal study protocols using the inExpose system (Gutor et al., 2024), mice were exposed once to SO_2_ at 125 ppm (vs. filtered air) for 4 h. Mice were subsequently treated with 50 μL of 1 μg of recombinant IL‐10 (50245‐MNAE, Sino Biological, Wayne, PA) or saline (vehicle) (volume) by intratracheal instillation for 3 doses over 2 days initiated 2–3 h post‐SO_2_ exposure (Figure 1a). Animals were euthanized (anesthetizing with isoflurane and then cervical dislocation) 5 h after the final IL‐10 treatment. Mice were weighed daily.

### 2.2 Serum, airway, and lung tissue collection

Serum was processed as previously described (Schwab et al., 2025). Bronchoalveolar lavage fluid (BALF) was collected using 3 × 1 mL of phosphate‐buffered saline (PBS). Total BALF cell counts from pooled lavages were enumerated using a BioRad TC 20 cell counter. Differential cell counts were determined from cytospin‐prepared slides (Cytopro Cytocentrifuge, ELITech Group, Logan, UT) stained with Diff‐Quick (Siemens, Germany). Cell‐free BALF (first lavage) was isolated for the measurement of protein mediators. After BALF isolation and removal of blood from pulmonary vasculature, lung tissue cells (1/2 of right lung lobes) and homogenates (1/2 right lung lobes) were prepared (Schwab et al., 2025) with left lung lobe processed for histology.

### 2.3 Soluble mediators and tissue injury markers

Serum pentraxin‐2 (MPTX20) and surfactant protein D (SP‐D) (MSFPDO) (R&D, Minneapolis, MN) and serum fibrinogen (MFG11‐K01, Eagle Bioscience, Inc., Amherst, NH) and lung fibronectin (ab108849, Abcam, Waltham, MA) were assessed using ELISA kits. Protein (BCA Protein Assay Kit, 23227, Thermo Scientific, Rockford, IL) and lactate dehydrogenase (LDH Assay Kit, ab102526, Abcam) were also assessed. IL‐6, IL‐10, CXCL1, CXCL2, CXCL10, CCL22, CCL4, CCL7, CCL8, and IL‐33 were assessed using a Luminex multiplex (LXSAMSM, R&D, Minneapolis, MN). The kits had lower limits of quantification of 0.159 ng/mL, 3.05 pg/mL, 25 ng/mL, 20 μg/mL, 1 U/mL, and for the Luminex 16.50, 158.68, 6.67, 18.81, 34.07, 65.84, 4.86, 9.92, 95.84, and 28.81 pg/mL, respectively. Substance P was assessed by ELISA (NBP3‐42246, Novus Biologicals, Centennial, CO) with a lower limit of quantification of 4.37 pg/mL. Serum samples were also analyzed via the VetScan VS2 Chemistry Analyzer using the Prep Profile II reagent rotors (500‐0026, Zoetis, Parsippany, NJ) for glucose, total protein, alanine aminotransferase (ALT), blood urea nitrogen (BUN), creatinine, and alkaline phosphatase.

### 2.4 Flow cytometry

Lung cell infiltrates were quantified following lung cell dissociation of 1/2 of each right lung lobe as previously described (Schwab et al., 2025), with total lung cells per animal enumerated using BioRad TC 20 cell counter. For immune cell analysis, single‐cell suspension was stained with LIVE/DEAD Fixable Dead Cell stain (L23105, BD Biosciences) followed by antibodies against mouse cell surface markers as previously described (Poole et al., 2025) (see also Table S1). Post‐acquisition, all flow cytometry data were exported and stored using the flow cytometry standard 3.1 format and subsequently analyzed using FlowJo software version 10.10.0 (FlowJo, Ashland, OR). The gating strategies for lung CD11c^−^Ly6G^+^Ly6C^+^ neutrophils, CD11c^hi^CD11b^var^ F4/80^+^ alveolar macrophages, CD11c^var^CD11b^var^CD103^+^ dendritic cells, CD11c^int^CD11b^+^CD103^−^Ly6C^−^MHC Class II^−^ resting interstitial macrophages (IM), CD11c^int^CD11b^+^CD103^−^Ly6C^−^MHC Class II^+^ activated IMs, CD11c^int^CD11b^+^CD103^−^Ly6C^+^ inflammatory IMs, CD11c^−^CD11b^+^Ly6C^+^ monocytic‐like cells, with gating strategy of these myeloid cells depicted in Figure S1, and lymphocytes were gated from the neutrophil negative and CD11c^−^CD11b^−^ gate for CD3^−^CD19^+^ B cells, CD3^+^CD4^+^ T cells, CD3^+^CD8^+^ T cells, CD3^−^NK1.1^+^ natural killer (NK) cells, all as previously reported (Mikuls et al., 2021; Nelson et al., 2018; Poole et al., 2012; Poole et al., 2017; Poole, Gaurav, et al., 2022; Robbe et al., 2015; Schwab et al., 2025). Lung cell populations were quantified by absolute counts by normalizing gated percentages to total cell numbers (multiplying percent gated of CD45^+^ cells by total lung cells).

### 2.5 Histopathology

Left lungs were excised and inflated to 15 cm H_2_O pressure with 10% formalin for 24 h to preserve pulmonary architecture as previously described (Poole et al., 2019). The fixed, paraffin‐embedded sections were cut (4 μm) at midpoint sections to include regions of large and small airways as well as blood vessels, with tissues stained for hematoxylin and eosin (H&E) or preserved for subsequent immunohistochemistry as previously described (Schwab et al., 2025). Lung sections were subsequently stained for apoptotic lung cells (C10618, Click‐iT Plus TUNEL assay, Invitrogen), neutrophils (anti‐myeloperoxidase/MPO, rabbit polyclonal, Abcam ab9535), anti‐peptidyl‐citrulline antibody (CIT, clone F95 IgMκ, #MABN328, Millipore Sigma), in‐house rabbit polyclonal IgG antibody to MAA (Poole, Mikuls, et al., 2022), and anti‐vimentin (#bs‐8533R‐Cy5, Bioss, Woburn, MA) with DAPI (4′6‐diamidino‐2‐phenylindole) (H‐1200) to identify nuclei as previously described (Johnson et al., 2023; Poole et al., 2025; Schwab et al., 2025). Immunofluorescent images were then captured using an Echo Revolve (Discover Echo, San Diego, CA) microscope. Expression of molecules was quantified (one lung section from each mouse/experimental group) with a common signal threshold of all lung fields per section at 40× magnification, using Image J (v2.0) software (NIH, Bethesda, MD) as previously described (Mikuls et al., 2021; Poole et al., 2025; Schwab et al., 2025).

### 2.6 Statistical analysis

Each experimental group included *n* = 20 mice with 10 males and 10 females to follow current recommendations to improve rigor, reproducibility, and generalizability of preclinical findings as well as allow for sex‐stratified analyses. Numbers less than the maximum number reflect limitations in the available sample quantity or quality. The Shapiro–Wilk test was utilized to test for normality among treatment groups. If the normality condition was satisfied, a one‐way ANOVA test was used, and if not satisfied, a Kruskal–Wallis test was used instead. Subsequent utilization of the two‐stage Benjamini, Krieger, and Yekutieli procedure was applied using a false discovery rate threshold of 5%. To determine sex differences, a two‐way ANOVA with a two‐stage linear step‐up procedure was utilized. All statistical analyses were performed using GraphPad Prism (version: 10.2.2) software, and statistical significance was accepted at a *p* value <0.05.

## 3 RESULTS

### 3.1 Lung‐targeted IL‐10 therapy reduced SO_2_ ‐induced weight loss and serum indices reflecting inflammation and lung injury

Lung‐delivered IL‐10 reduced SO_2_‐induced weight loss at Day 2 relative to vehicle treatment for both females and males, and when combined, there was less weight loss at day 2 in mice treated with IL‐10 (14.1% body weight loss) compared to animals treated with vehicle (19.2% body weight loss; *p* = 0.0066, Figure 1b). There was no mortality.

Serum levels of pentraxin‐2 and fibrinogen (acute phase reactants), total protein and SP‐D (markers of lung permeability, disruption of barrier function), and LDH (marker of injury/cell death) induced by SO_2_ were reduced with IL‐10 treatment (Figure 1c). Additionally, IL‐10 attenuated decreases in blood glucose levels (marker of glucose consumption) induced by SO_2_ (Figure 1c), a phenomenon also observed with endotoxin exposure and IL‐10 treatment (Poole, Gaurav, et al., 2022). There were otherwise no differences in other serum chemistry indices including alanine aminotransferase, blood urea nitrogen, and creatinine across all treatment groups, with the exception that SO_2_‐induced decreases in serum alkaline phosphatase were not modulated by IL‐10 treatment (Table S2).

Sex‐based differences were demonstrated for specific outcomes including significantly increased levels of serum pentraxin‐2 (*p* = 0.0129), fibrinogen (*p* = 0.0143), and LDH (*p* = 0.0063) (but not serum total protein, SP‐D, and glucose) in male versus female mice treated with SO_2_ + vehicle (Figure S2a). Serum LDH levels were higher (*p* = 0.0015) in male sham versus female sham mice (Figure S2a), an observation previously reported by others and explained by multiple physiological, metabolic, and genetic factors (e.g., higher tissue mass and glycolytic activity) attributed to testis‐specific isoenzymes (Mazzaccara et al., 2008; Odet et al., 2008; Wang et al., 2025).

### 3.2 IL‐10 treatment decreases several SO_2_ ‐induced inflammatory, injury, and tissue repair mediators in the lung

**FIGURE 2:**
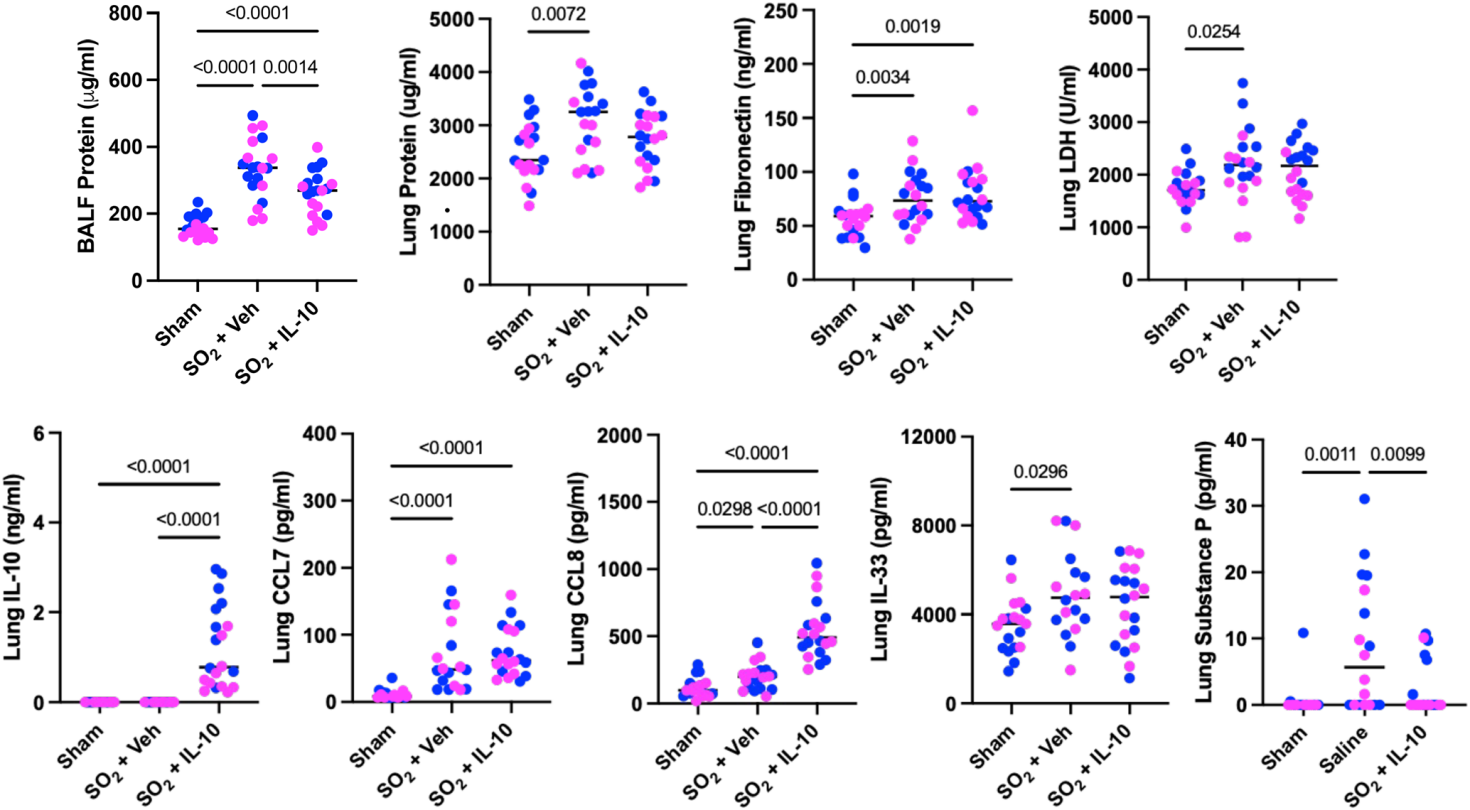
IL‐10 treatment decreases several SO_2_‐induced inflammatory, injury, and wound repair mediators in the lung. BALF and lung tissue homogenates utilized to quantify protein levels. Lung levels of fibronectin, lactate dehydrogenase (LDH), IL‐10, CCL7, CCL8, IL‐33, and Substance P were also quantified. All data expressed as mean of *n* = 20 mice/group (10 female, magenta; 10 male, blue), with values less than 20 due to limited sampling. Statistically significant *p* values denoted by line.

To characterize the effect of IL‐10 therapy on modulating lung inflammatory, injury, and tissue repair processes, BALF and lung tissues were assessed for specific mediators (Figure 2). As a marker of alveolar‐capillary barrier permeability, BALF protein levels were increased following SO_2_ exposure as compared to sham, and this response was reduced with IL‐10. Lung tissue total protein, a generalized marker of inflammatory lung responses, was increased with SO_2_ + vehicle, but not SO_2_ + IL‐10, versus sham. Lung levels of fibrinogen, a marker of injury and repair, were increased with SO_2_ exposure with no difference between vehicle and IL‐10 treatment. Lung levels of LDH (cell injury/death) were significantly increased with SO_2_ + vehicle (*p* = 0.0254) versus sham and trended (*p* = 0.055) higher for SO_2_+ IL‐10 treatment versus sham. Lung IL‐10 levels were detected only in mice treated with IL‐10, with levels ranging from 0.22 to 2.96 ng/mL at time of necropsy, 5 h following final IL‐10 treatment. Of the chemokines/cytokines investigated, SO_2_ induced increases in CCL7, CCL8, and IL‐33 but not CXCL1 and IL‐6 (Figure 2 and Table S2). In the SO_2_ + IL‐10 treatment group vs. sham, there were increased levels of CCL8, and this response was also significantly increased with SO_2_ + IL‐10 as compared to SO_2_ + vehicle treatment. There was no significant increase in IL‐33 in SO_2_ + IL‐10 vs. sham (*p* = 0.11). Levels of TNF‐⍺ were not detected (data not shown). SO_2_ also induced increases in the neuropeptide Substance P, a response that was significantly (*p* = 0.0099) reduced with IL‐10 treatment.

Sex‐based differences were demonstrated for lung LDH, IL‐10 levels, and Substance P (Figure S2b). Lung LDH levels were increased in male vs. female mice for both SO_2_ + vehicle (*p* = 0.008) and SO_2_ + IL‐10 (*p* = 0.006). Lung IL‐10 levels in SO_2_ + IL‐10 treated mice were increased in males vs. females (mean ± SD, 1.74 ± 0.94 ng/mL vs. 0.67 ± 0.52 ng/mL, respectively, *p* < 0.0001). Lung Substance P levels were increased in SO_2_ + vehicle male versus female mice (*p* = 0.0072).

### 3.3 Lung‐targeted IL‐10 treatment reduces SO_2_ ‐induced airway and lung cell infiltrates

**FIGURE 3:**
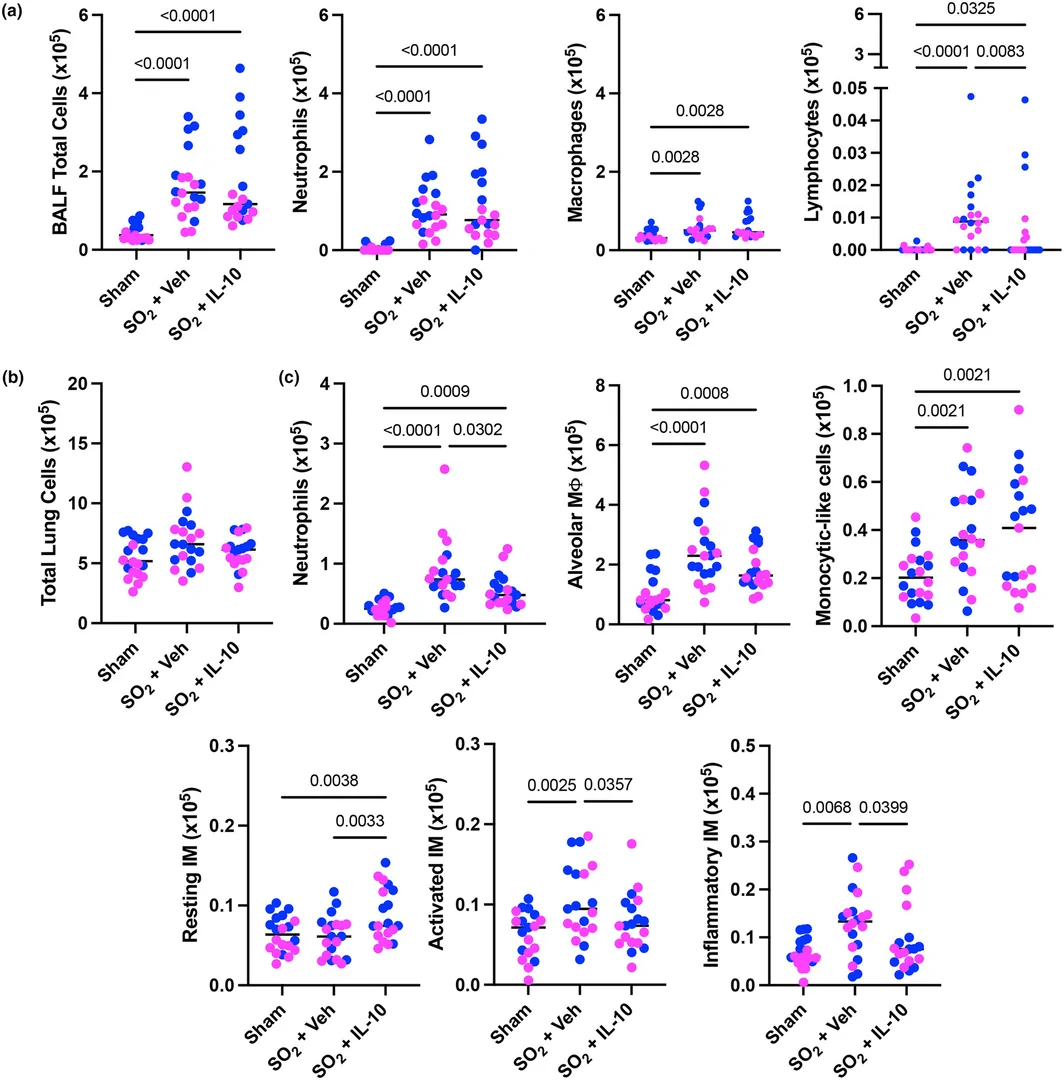
Lung‐targeted IL‐10 treatment reduces SO_2_‐induced airway and lung cell infiltrates. (a) BALF total cell, neutrophil, macrophage, and lymphocyte numbers across indicated groups. (b) Total lung cells of mice across treatment groups were enumerated. (c) Lung immune cell infiltrates across groups determined by flow cytometry on live CD45^+^ cells after exclusion of debris, doublets with lung cell % population in respective gate multiplied by total lung cells. Cells defined as CD11c^−^Ly6G^+^LyC6^+^ neutrophils, CD11c^hi^F4/80^+^CD11b^var^ alveolar macrophages, CD11c^−^CD11b^+^Ly6C^+^ monocytic‐like cells, CD11c^int^CD11b^+^CD103^−^Ly6C^−^MHC Class II^−^ resting interstitial macrophages (IM), CD11c^int^CD11b^+^CD103^−^Ly6C^−^MHC Class II^+^ activated IM, and CD11c^int^CD11b^+^CD103^−^Ly6C^+^MHC Class II^−^ inflammatory IM. All data expressed as mean of *n* = 20 mice/group (10 female, magenta; 10 male, blue), with values less than 20 due to limited sampling. Statistically significant *p* values denoted by line.

SO_2_‐induced airway influx of lymphocytes, but not total cells, neutrophils, and macrophages, was significantly reduced in the lavage fluid with IL‐10 versus vehicle treatment (Figure 3a). There were again significant sex‐based differences, with male mice exhibiting increased numbers of lavage fluid total cells (SO_2_ + vehicle, *p* = 0.009 and SO_2_ + IL‐10, *p* < 0.0001), neutrophils (SO_2_ + vehicle, *p* = 0.01 and SO_2_ + IL‐10, *p* < 0.0001), and macrophages (SO_2_ + IL‐10, *p* = 0.0005) (but not lymphocytes) as compared to female mice following respective treatment conditions (Figure S2c).

There was no difference in lung tissue total cells enumerated across treatment groups (Figure 3b); however, differences in specific lung cells as identified by flow cytometry were demonstrated (Figure 3c). SO_2_ exposure significantly induced recruitment of Ly6G^+^Ly6C^+^ neutrophils, CD11c^hi^F4/80^+^CD11b^var^ alveolar macrophages, CD11c^−^CD11b^+^Ly6C^+^ monocytic‐like cells, CD11c^int^CD11b^+^CD103^−^LyC‐MHC Class II^+^ activated interstitial macrophages (IM), and CD11c^int^CD11b^+^CD103^−^Ly6C^+^MHC Class II^−^ inflammatory IMs. As compared to vehicle control, IL‐10 treatment significantly reduced SO_2_‐induced recruitment of neutrophils, activated IMs, and inflammatory IMs. Conversely, IL‐10 treatment following SO_2_ exposure resulted in increased CD11c^int^CD11b^+^CD103^−^Ly6C^−^MHC Class II^−^ resting IMs as compared to both sham and SO_2_ + vehicle treatment conditions. There were no differences across treatment groups in numbers of CD19^+^ B cells, CD3^+^CD4^+^ T cells, CD3^+^CD8^+^ T cells, CD3^−^NK1.1^+^ natural killer cells, and CD11c^var^CD11b^var^CD103^+^ dendritic cells (Table S2).

There were significant sex‐based differences in lung cell infiltrates (Figure S2d) with male sham mice demonstrating an increased number of total lung cells versus female sham mice (*p* = 0.0052). However, there were increased numbers of lung neutrophils (*p* = 0.0195, SO_2_ + vehicle) and inflammatory IMs (*p* = 0.0251, SO_2_ + IL‐10) in female versus male mice, and there were increased numbers of resting IMs (*p* = 0.0213, sham) and monocytic‐like cells (*p* = 0.041, SO_2_ + IL‐10) in male versus female mice. No other sex‐based differences were demonstrated in the other lung cell infiltrates investigated (data not shown).

### 3.4 IL‐10 treatment restores lung homeostasis by reducing SO_2_ ‐induced lung cell apoptosis, and expression of MAA and CIT modified proteins and vimentin

**FIGURE 4:**
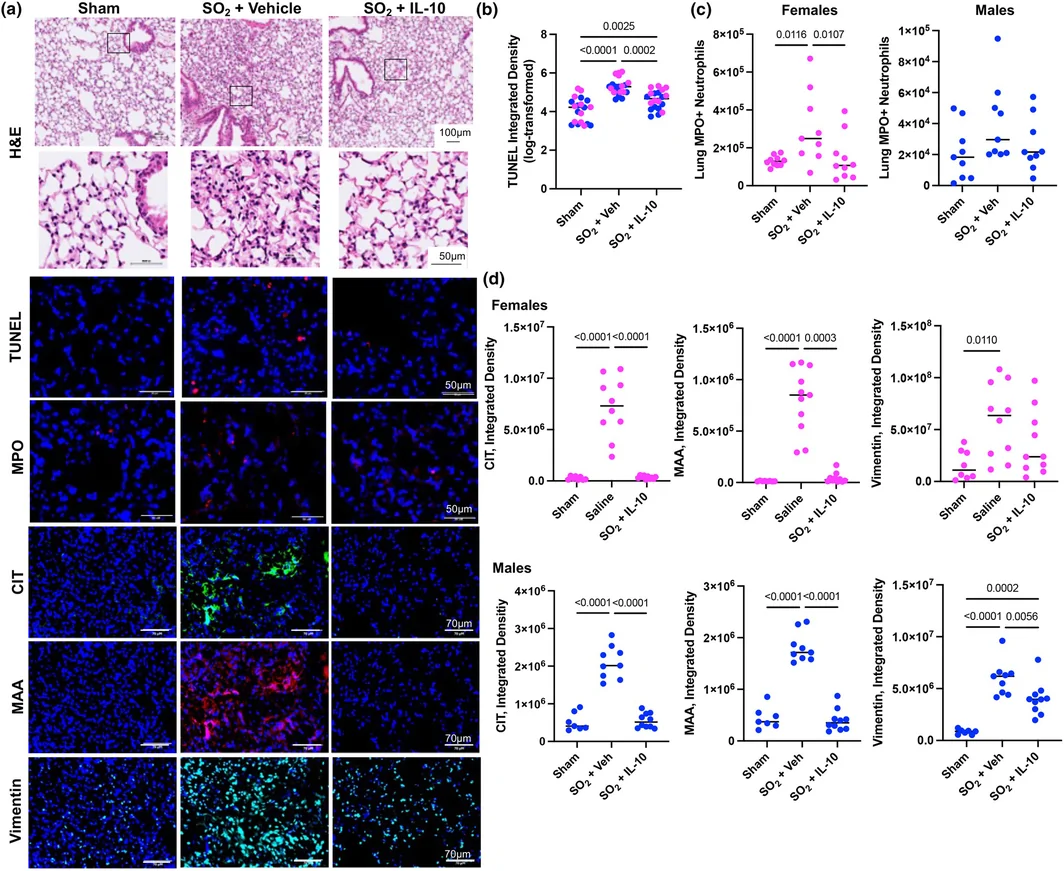
IL‐10 treatment restores lung homeostasis by reducing SO_2_‐induced lung cell apoptosis, lung neutrophils, and expression of MAA and CIT modified proteins and vimentin production. (a) Representative H&E (10× and 40× of insert)‐stained lung sections and confocal microscopy images of TUNEL‐positive cells (apoptotic cells, red), myeloperoxidase (MPO)^+^ neutrophils, citrulline (CIT, teal) and malondialdehyde‐acetaldehyde (MAA, red) modified proteins and vimentin (teal) with nuclei (DAPI, blue)‐stained lung sections from each treatment group. Scatter dot plots depict means of integrated density of lung tissue TUNEL‐positive cells (b), neutrophils (c), and CIT, MAA, and vimentin expression (d). All data expressed as mean with SD bars of *n* = 10 female (magenta) and *n* = 10 male (blue) mice/group with values less than 10 due to limited sampling. Statistically significant *p* values denoted by line.

Representative lung tissue staining for H&E, TUNEL, MPO, CIT, MAA, and vimentin are shown (Figure 4a). In H&E‐stained lung tissues, there were diffuse increases in lung cellular infiltrates with SO_2_ exposure. SO_2_ exposure increased TUNEL‐positive cells in lung tissue, indicating enhanced apoptosis, which was significantly reduced (*p* = 0.0002) in mice receiving IL‐10 treatment (Figure 4b). There were no sex‐based differences with TUNEL‐positivity across treatment conditions. SO_2_ induced increased lung MPO^+^ neutrophils in female but not male mice, and this response was decreased (*p* = 0.0107) with IL‐10 treatment in female mice (Figure 4c).

Based upon prior findings that environmental microbial components including endotoxin induce post‐translational changes implicated in inflammatory disease (Schwab et al., 2025; Schwab, Wyatt, Moravec, et al., 2024), lung tissues were stained and quantified for CIT‐ and MAA‐modified antigens as well as vimentin, a type III intermediate filament protein that is commonly targeted by post‐translational modifications and involved in tissue repair (Figure 4a,d). CIT‐ and MAA‐modified proteins and vimentin were significantly increased following SO_2_ exposure versus sham in both female and male mice, but of differing magnitudes, with CIT and vimentin increased more in female (vs. male) mice (*p* < 0.0001 and *p* < 0.0001, respectively) and MAA increased to a greater degree in male (vs. female) mice (*p* < 0.0001). Moreover, there were striking reductions in expression of CIT‐ and MAA‐modified proteins with IL‐10 treatment (vs. vehicle), in both female and male mice. Treatment with IL‐10 (vs. vehicle) resulted in a reduction in SO_2_‐induced vimentin, a difference that was significant in male mice but that did not achieve significance in female mice.

## 4 DISCUSSION

Inhalant hazardous chemicals represent high‐consequence public health threats for which effective post‐exposure interventions are lacking (Marzec & Nadadur, 2024). Previous preclinical studies demonstrated that lung‐targeted IL‐10 treatment, administered after exposure, robustly reduced multiple lung and systemic inflammatory indices induced by one‐time and repeated exposure to high‐concentration endotoxin as well as one‐time exposure to high‐concentration, microbial‐component‐enriched agricultural organic dust extracts (Poole, Gaurav, et al., 2022; Schwab et al., 2025; Schwab, Wyatt, Nelson, et al., 2024). Here, proof‐of‐concept, preclinical animal studies inform the potential beneficial translatability of intratracheally administered IL‐10 treatment following a one‐time exposure to high concentration SO_2_, an important chemical implicated in toxin‐associated lung disease. Specifically, short‐term, intratracheally administered IL‐10 treatment reduced SO_2_‐induced weight loss, systemic inflammation, lung barrier dysfunction, cell toxicity, tissue repair mediators, neuropeptide expression, inflammatory lung cell recruitment, lung vimentin deposition, and post‐translationally modified protein expression. These findings support future studies toward developing lung‐delivered IL‐10 as a potential post‐exposure therapy to reduce adverse consequences of acute lung injury and inflammation in a broader context that includes chemicals/toxins.

Hallmarks of acute lung injury and its severe form of acute respiratory distress syndrome across most etiologies generally include lung tissue damage, increased epithelial cell permeability, inflammatory cell recruitment and mediator release, surfactant and host immune dysfunction, oxidative stress, impaired gas exchange, and lung dysfunction (Wick et al., 2024). However, little is known about the acute, nonfatal lung effects beyond 1 day after exposure to high‐concentration SO_2_ exposure. These current studies focused on 2 days following a one‐time, high‐concentration exposure to SO_2_ (125 ppm × 4 h), and demonstrated that this model recapitulates several key features of acute lung injury. These included systemic and lung responses consistent with ongoing tissue injury and inflammation including weight loss, acute phase protein reactant production, altered glucose metabolism, epithelial barrier disruption, neurogenic inflammation, and induction of mediators associated with leukocyte recruitment and tissue repair. The increases in neutrophils, macrophages, and specific lung myeloid cell subpopulations further suggest the establishment of a coordinated innate immune response that persists beyond the immediate post‐exposure period. Likewise, increased apoptosis and accumulation of CIT‐ and MAA‐modified proteins indicate that oxidative and post‐translational protein modification may contribute to tissue injury and remodeling following SO_2_ exposure. Collectively, these findings support the utility of this model for investigating disease pathogenesis and mechanisms induced by chemical injury and serve to test therapeutics.

Importantly, short‐term, intratracheally administered IL‐10 significantly reversed several adverse systemic and lung consequences induced by acute SO_2_ exposure. The IL‐10/IL‐10R‐α signaling cascade is mediated by the Jak1/Tyk2/STAT3 pathway (Carlini et al., 2023), leading to suppression of MAPKs and NF‐kB pathways and transcriptional repression of pro‐inflammatory cytokines (Lee et al., 2014; Lehmann et al., 2005; Wyatt et al., 2020), collectively promoting anti‐inflammatory and pro‐resolving effects of IL‐10. These established signaling mechanisms provide a possible explanation for the broad protective effects observed in this study. Consistent with these activities, IL‐10 treatment attenuated SO_2_‐induced disruption of barrier integrity, cell death, apoptosis, inflammatory cell infiltrates, neuropeptide expression, and expression of post‐translationally modified proteins. Together, these findings suggest that inflammatory and oxidative pathways are important contributors to the injury response in this model and are responsive to IL‐10 treatment.

Although the present study was not designed to define precise molecular mechanisms responsible for IL‐10‐mediated protection following SO_2_ exposure, several observations are consistent with previously described actions of IL‐10. For example, IL‐10 signaling can activate STAT3‐dependent pathways that promote cell survival through Bcl‐2 family signaling and reduced Fas/FasL‐ and TNF‐mediated apoptosis signaling (Carlini et al., 2023), which may contribute to the reduced apoptosis observed here. Moreover, IL‐10's ability to limit early pro‐inflammatory mediators and chemokines (Schwab et al., 2025) might explain the reduction in SO_2_‐induced recruitment of monocyte/macrophage subpopulations and neutrophils. IL‐10 can influence tissue redox balance by limiting the production of inflammatory cytokines and release of free oxygen radicals and nitrites (Haddad & Fahlman, 2002). Consistent with this possibility, IL‐10 treatment markedly reduced CIT‐ and MAA‐modified proteins in this current study. Future mechanistic studies will be important to define specific pathways through which IL‐10 mitigates SO_2_‐induced lung injury and inflammation.

This current study provides information on sex‐based differences in response to acute chemical SO_2_ exposure. Whereas the observed sex‐dependent differences were generally modest, they suggest that biological sex may influence both the lung response to SO_2_ injury and response to inhaled IL‐10 therapy. Sex‐specific differences in innate immune signaling, oxidative stress responses, and tissue repair pathways have been widely reported in experimental lung injury models and human acute lung injury, and are influenced, at least in part, by sex hormones (Cabello et al., 2015; Card et al., 2006; Lemos‐Filho et al., 2013; McNicholas et al., 2019; Ray & Holian, 2019; Zhao et al., 2025). Estrogen signaling has been associated with modulation of neutrophil trafficking, macrophage phenotype, antioxidant defenses, and cytokine production, and androgens can alter inflammatory and immune regulatory pathways (Chakraborty et al., 2023; Hoffmann et al., 2023; Kadel & Kovats, 2018). These mechanisms may contribute to the inflammatory signatures demonstrated here, including the heightened systemic inflammatory consequences and expression of MAA‐modified proteins in males, and the greater lung neutrophil burden, CIT‐modified proteins in females. A noteworthy observation was that lung IL‐10 levels recovered from male mice were much higher than those of females. Although sex‐based differences in aerosol deposition attributed to greater lung volumes, airway diameters, tidal volumes, and inspiratory capacity have been recognized (Barnthaler et al., 2024; Kolanjiyil et al., 2019), it is also possible that male and females differ in endogenous IL‐10 receptor signaling, cytokine retention, or clearance. In addition, SO_2_‐induced bronchoconstriction could potentially alter intrapulmonary distribution of subsequently administered therapeutics, and thus additional studies are needed to evaluate clinically relevant delivery modalities, aerosol deposition, dose distribution, and therapeutic effectiveness under conditions that more closely reflect real‐world exposure scenarios.

Although no adverse effects were observed within the study timeframe, the possibility of delayed or cumulative immunosuppressive effects with long‐term IL‐10 administration cannot be excluded. We previously demonstrated that there were no differences between three repeated doses (short term) of IL‐10 only and vehicle control for numerous outcomes, including weight changes, serum systemic inflammatory markers, BALF counts, lung cell infiltrates, lung pathology, and the presence of post‐translationally modified proteins including MAA and CIT (Schwab et al., 2025). However, the short half‐life of IL‐10 (4–6 h) (Minshawi et al., 2020) may necessitate repeated dosing to sustain anti‐inflammatory effects in repeated exposure settings; however, this pharmacokinetic property may also represent a safety advantage, permit rapid termination of biological activity, and limit prolonged immune modulation. Furthermore, lung‐localized delivery may further enhance safety by minimizing systemic exposure. Future studies will be needed to investigate longer‐term exposures with repeated SO_2_ and IL‐10 dosing to determine relevant treatment effects.

There are limitations to this study. Whereas invasive lung function measurements of airway resistance, compliance, and hyperresponsiveness (AHR) were assessed at Day 2 using a computerized small‐animal ventilator system, there were no differences in airway mechanics detected among the three treatment groups (Sham, SO_2_ + vehicle, SO_2_ + IL‐10) (data not shown). This may reflect recovery rather than ongoing injury, as SO_2_‐induced bronchoconstriction occurs rapidly and typically resolves within hours. The SO_2_ exposure paradigm was intentionally constrained to avoid excessive morbidity and mortality; however, this resulted in a relatively modest lung injury and inflammatory responses at 2 days post‐exposure, which may have limited the detectable magnitude of therapeutic benefit. IL‐10 treatment was also initiated 2–3 h after exposure to model a clinically relevant delay rather than immediate prophylaxis, and it remains possible that earlier administration could confer greater benefit.

Overall, these findings support further investigation of lung‐targeted IL‐10 delivery as a potentially translatable strategy for mitigating lung injury following extreme environmental exposures, including chemical insults represented by SO_2_ exposure. This work lays a foundation for future studies to define the broader applicability of IL‐10‐based interventions across additional high‐consequence exposure scenarios and other etiologies of acute lung injury, such as infectious, allergic, and aspiration‐related inflammation.

## AUTHOR CONTRIBUTIONS

**Amy J. Nelson:** Conceptualization; data curation; formal analysis; investigation; methodology; project administration; validation; visualization. **Todd A. Wyatt:** Conceptualization; investigation; methodology; project administration; resources; supervision; validation; visualization. **Bradley W. Richmond:** Conceptualization; investigation; methodology; visualization. **Belinda Diaz:** Data curation; investigation; methodology; visualization. **Angela M. Gleason:** Investigation; methodology; validation; visualization. **Michael J. Duryee:** Data curation; formal analysis; investigation; validation; visualization. **Geoffrey M. Thiele:** Data curation; formal analysis; investigation; methodology; resources; validation; visualization. **Ted R. Mikuls:** Data curation; formal analysis; investigation; methodology; project administration; resources; supervision; validation; visualization. **Jill A. Poole:** Conceptualization; data curation; formal analysis; funding acquisition; investigation; methodology; project administration; resources; software; supervision; validation; visualization.

## FUNDING INFORMATION

The main funding support is from a grant by the National Institute of Occupational Safety and Health (R01OH012045; JAP). JAP and TRM are also funded by the Department of Defense, PR200793. Other funding support includes VA (BLR&D Merit I01 BX005886, TAW, and CSR&D Merit I01 CX002995, TRM), National Institute of Health (2U54GM115458, TRM), and Rheumatology Research Foundation (TRM). TAW is the recipient of a Research Career Scientist Award (IK6 BX005962).

## CONFLICT OF INTEREST STATEMENT

JAP is a site recruiter for clinical industry studies for asthma, sinus disease, and mastocytosis (GlaxoSmithKline, AstraZeneca, Regeneron Pharmaceuticals, Blueprint Medicine). TRM has consulted for Horizon Therapeutics (Amgen), Otaltec Therapeutics, Pfizer, and UCB and has received research support from Horizon; royalties from Elsevier and Wolters Kluwer (UpToDate).

## ETHICS STATEMENT

All animal procedures were approved by the Institutional Animal Care and Use Committee (IACUC) and were conducted in accordance with relevant national and institutional guidelines for the care and use of laboratory animals.

## Supporting information


Figures S1–S2.

Tables S1–S2.

## Data Availability

Source data for this study are openly available at https://doi.org/10.5281/zenodo.14151997.
