# Supplementary material for: Intratracheal interleukin‐10 administration reduces acute lung injury induced by sulfur dioxide exposure in mice
Source: Physiol Rep. 2026 Aug 25;14(16):e71079. doi: 10.14814/phy2.71079 (PMC13507516; doi:10.14814/phy2.71079)
Supplement: Supplementary file 1 [file PHY2-14-e71079-s001.docx]

**Supplemental Material**

| **Supplemental Table S1.** Table of flow cytometry antibodies. | | | |  |
| --- | --- | --- | --- | --- |
| **Antigen** | **Species** | **Clone** | **Vendor** | **Fluorophore** |
| CD45 | Rat anti-mouse | 30-F11 | BD Biosciences (563053) | BV605 |
| CD11b | Rat anti-mouse | M1/70 | BD Biosciences (550993) | PerCP-Cy5.5 |
| Ly6G | Rat anti-mouse | 1A8 | BD Biosciences (561236) | AF700 |
| CD11c | Rat anti-mouse | N418 | Invitrogen  (61-0114-82) | PE-eFluor610 |
| F4/80 | Rat anti-mouse | T45-2342 | BD Biosciences (565614) | BUV 395 |
| Ly6C | Rat anti-mouse | HK1.4 | BioLegend (128041) | BV785 |
| MHC Class II | Rat anti-mouse | M5/114.15.2 | Cell Signaling  (664776S) | APC |
| CD103 | Armenian Hamster-anti-mouse | 2E7 | Invitrogen  (367-1031-82) | BUV737 |
| CD4 | Rat anti-mouse | RM4-5 | BD Biosciences (553047) | FITC |
| CD8a | Rat anti-mouse | 53-6.7 | BD Biosciences (553033) | PE |
| CD19 | Rat anti-mouse | 1D3 | Invitrogen  (47-0193-82) | APC-eFluor780 |
| CD3e | Hamster anti-mouse | 145-2C11 | BD Biosciences (552774) | PE-Cy7 |
| NK1.1 | Mouse anti-mouse | PK136 | BD Biosciences (550627) | APC |

**Supplemental Table 2.** Serum chemistries, lung cytokines/chemokines and lung cellular responses not modulated by IL-10 across treatment groups.

|  | Sham | SO_2_ + Vehicle | SO_2_ + IL-10 |
| --- | --- | --- | --- |
| **Serum** |  |  |  |
| ALT (U/L) | 31.0 ± 3.8 | 31.9 ± 5.7 | 28.8 ± 8.0 |
| BUN (mmol/L) | 8.7 ± 1.2 | 8.1 ± 1.21 | 7.7 ± 1.6 |
| Creatinine (μmol/L) | 18.9 ± 2.6 | 18.1± 0.3 | 18.6± 1.4 |
| Alkaline Phosphatase (U/L) | 97.8 ± 16.0 | **66.3 ± 15.8^*^** | **66.3 ± 13.1^*^** |
| **Lung Cytokines/Chemokines** |  |  |  |
| CXCL1 (pg/ml) | 356 ± 142 | 299 ± 149 | 322± 147 |
| IL-6 (pg/ml) | 187 ± 48 | 149 ± 38 | 166 ± 57 |
| **Lung Cells, x10^5^** |  |  |  |
| CD19^+^ B cells | 1.27 ± 0.39 | 1.22 ± 0.45 | 1.13 ± 0.37 |
| CD3^+^CD4^+^ T cells | 0.28 ± 0.14 | 0.24 ± 0.11 | 0.30 ± 0.12 |
| CD3^+^CD8^+^ T cells | 0.23 ± 0.13 | 0.19 ± 0.11 | 0.21 ± 0.07 |
| Natural Killer cells | 0.26 ± 0.19 | 0.16 ± 0.0.7 | 0.23 ± 0.13 |
| Dendritic cells | 0.017 ± 0.010 | 0.023 ± 0.017 | 0.017 ± 0.008 |

Mean ± SD. Statistical difference vs. sham (^*^p<0.0001) and **bold.**

Serum studies: n=10 female and male mice/group; Lung studies: n=18-20 female and male mice/group.

No significant detection above lower limit of detection for CCL4, CCL22, CXCL2, CXCL10, and CXCL22.

ALT: Alanine aminotransferase; BUN: Blood urea nitrogen
