# Supplementary material for: Intratracheal interleukin‐10 administration reduces acute lung injury induced by sulfur dioxide exposure in mice
Source: Physiol Rep. 2026 Aug 25;14(16):e71079. doi: 10.14814/phy2.71079 (PMC13507516; doi:10.14814/phy2.71079)
Supplement: Supplementary file 2 [file PHY2-14-e71079-s002.zip › Supplemental Figures 1 and 2.docx]

**Supplemental Figure 1. Gating strategy for identification of non-debris, singlets, live CD45^+^ cells and myeloid cell subpopulations.** For flow analysis, all panels were first gated as forward scatter-area (FSC-A) x side scatter-area (SSC-A) to omit debris, dead or apoptotic cells. This was followed by two single-cell gates to omit doublets (FSC-A x FSC-height (H) and SSC-A x SSC-H), followed by live/dead, and then CD45 gate to assure removal of any additional dead or apoptotic cells and non-leukocytes. The CD45^+^ cells were placed on a CD11c x Ly6G gate and then Ly6C x Ly6G gate to select neutrophils. Non-neutrophils were placed on CD11c x CD11b gate to identify macrophages, monocytes, and dendritic cells. Specifically, CD11c^hi^CD11b^variable^ cells were then gated by F4/80 x CD11b to identify CD11c^hi^CD11b^var^F4/80^+^ alveolar macrophages. CD11c^int^CD11b^var^ cells were placed on CD103 x FSC-A gate to identify CD11c^int^CD11b^var^CD103^+^ dendritic cells. CD103-negative cells were placed on Ly6C x MHC Class II gate to identify CD11c^int^CD11b^+^CD103^-^Ly6C^-^MHC class II^-^ resting interstitial macrophages (IM), CD11c^int^CD11b^+^CD103^-^Ly6C^+^MHC class II^-^ inflammatory IM, and CD11c^int^CD11b^+^CD103^-^Ly6C^-^MHC class II^+^ activated IM. The CD11c^-^CD11b^+^ cells from non-neutrophil gate were placed on Ly6C x FSC-A gate to identify CD11c^-^CD11b^+^Ly6C^+^ monocytic-like cells.

**Supplemental Figure 2. Sex-based significant differences demonstrated for specific experimental outcomes.** Scatter dot-plots depict mean of sham (black), SO_2_ + vehicle (purple), and SO_2_ + IL-10 treatment (orange). **A,** Serum levels of pentraxin-2, fibrinogen, and lactate dehydrogenase (LDH). **B**, Lung homogenate levels of LDH, IL-10, and Substance P. **C,** Bronchoalveolar lavage fluid (BALF) levels of total cells, neutrophils, and macrophages. **D,** Total lung cell infiltrates with subpopulations of CD11c^-^Ly6G^+^LyC6^+^ neutrophils, CD11c^-^ CD11c^int^CD11b^hi^CD103^-^Ly6C^-^MHC Class II^-^ resting interstitial macrophages (IM), CD11c^int^CD11b^hi^ CD103^-^Ly6C^+^ inflammatory IM, and CD11b^+^Ly6C^+^ monocytic-like cells. N=10/mice group, with values less than 10 due to limited sampling. Statistically significant p-values denoted by line.
